# A Comprehensive Review of Technological Advances in Meat Safety, Quality, and Sustainability for Public Health

**DOI:** 10.3390/foods15010047

**Published:** 2025-12-23

**Authors:** Abdul Samad, Ayesha Muazzam, A. M. M. Nurul Alam, SoHee Kim, Young-Hwa Hwang, Seon-Tea Joo

**Affiliations:** 1 Division of Applied Life Science (BK21 Four), Gyeongsang National University, Jinju 52828, Republic of Korea; buzdarabdulsamad@gmail.com (A.S.); ashu2nice@gmail.com (A.M.); alam6059@yahoo.com (A.M.M.N.A.); winight123@gmail.com (S.K.); 2 Institute of Agriculture & Life Science, Gyeongsang National University, Jinju 52828, Republic of Korea

**Keywords:** meat safety, sustainability, alternative proteins, public health, technological innovations

## Abstract

The demand for food is increasing with the rise in the human population. Among foods, meat is an essential part of human nutrition. Meat provides good-quality protein and all the micronutrients needed by humans. In addition, it also contains some bioactive compounds that are good for human health. Increasing demand, together with concerns over food safety, requires new approaches to guarantee a sustainable, safe, and healthy meat supply chain. The only way to get over these challenges is through technological innovations that are capable of enhancing the safety, quality, and sustainability of meat. Herein, this review identifies the need for new methods of rapid microbial detection, biosensors, AI-based monitoring, innovative processing and preservation techniques, precision livestock farming, resource-efficient feed and water management, alternative protein sources, and circular economy approaches. In particular, this review examines some meat analogs like cultured meat, hybrid products, and microbial proteins as environmentally friendly and nutritionally balanced alternatives. These changes in technology can also bring benefits to consumers in terms of their health. The health benefits of these technological innovations for consumers go beyond just safety, including improved nutritional profiles, functional bioactive ingredients, and the prevention of antimicrobial resistance. The review further analyzes policies, regulatory frameworks, and ethical considerations necessary to achieve consumer trust and social acceptance, including the global alignment of standards, certification, labeling, and all issues related to ethics. Furthermore, AI, IoT, Big Data, and nutritional technologies represent new emerging trends able to unleash new opportunities for the optimization of production, quality control, and personalized nutrition.

## 1. Introduction

Since the beginning of human history, meat has been a vital part of our diet, providing numerous essential nutrients that are difficult to obtain from other food sources [1]. Meat is a high-biological-value protein source, containing essential amino acids, which are necessary for the growth, maintenance, and repair of lean tissue [2]. Meat is considered a readily bioavailable source of heme iron, significantly improving the body’s iron status [3]. The meat is also a source of vitamins (e.g., B12, a micronutrient essential for neurological development and hematological activity) [4]. Moreover, meat is also the source of zinc, selenium, phosphorus, some B-complex vitamins, and long-chain omega-3 fatty acids [5].

**Figure 1:**
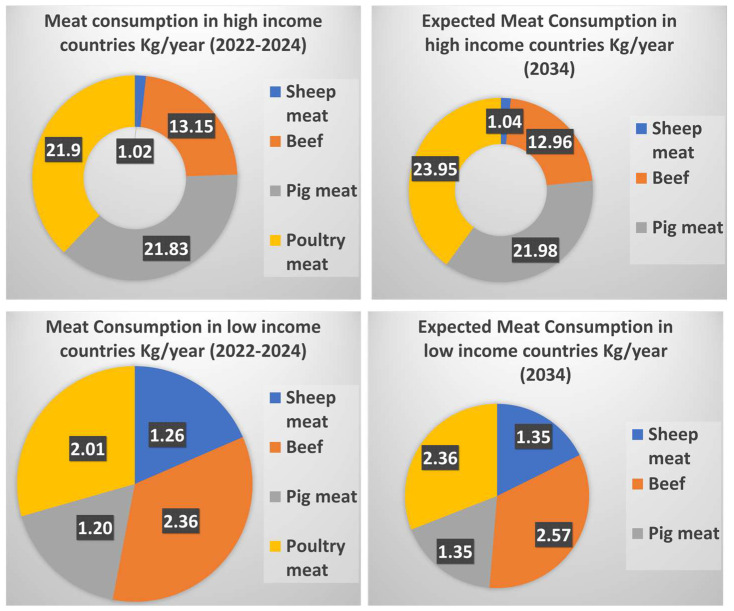
Species-wise per-capita meat consumption trends in low- and high-income countries (2012–2034), expressed as kg/person/year) (Modified from OECD–FAO [9]).

Along with its nutritional value, meat has high digestibility levels, which directly improve protein quality measures in diets [6]. At the population level, patterns of meat consumption have undergone significant changes in tandem with socioeconomic development [7]. As a result, global per capita meat consumption increased steadily through the second half of the 20th century and into the early 21st century, especially in many low- and middle-income countries [8]. It is projected that global meat demand will continue to evolve over the coming decade, driven by increased income, urbanization, and shifting consumer preferences, and this trend is further elaborated in Figure 1.

**Figure 2:**
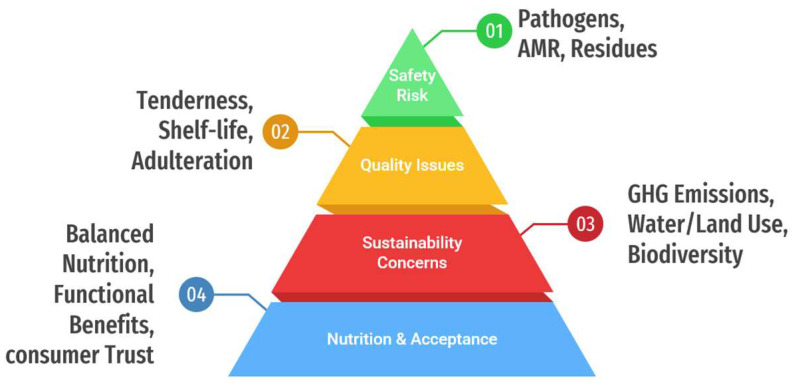
Key dimensions of risk in animal-source food production and consumption.

Collectively, these nutritional and socioeconomic factors underscore the central importance of meat. Public health, agricultural policy, and technological innovation collectively aim to ensure that meat supplies remain safe, sustainable, and nutritionally adequate [10]. Despite the nutritional benefits of meat, the meat sector faces persistent and complex challenges across safety, quality, and sustainability domains that threaten public health and system resilience [11,12]. The significant risks associated with animal-based food production and consumption are illustrated in Figure 2. From a food safety standpoint, meat and meat products are particularly vulnerable to the transmission of bacterial, viral, and parasitic pathogens [13]. Global estimates of foodborne disease indicate that bacterial pathogens cause significant illness and mortality worldwide [14]. Many of these bacteria are linked to foods of animal origin [15]. Outbreaks of these bacteria can still occur in retail environments [16], food processing facilities [17], and even home kitchens [18]. Concomitantly, the emergence of antimicrobial-resistant (AMR) bacteria associated with food animal production has become a significant problem from a One Health perspective. Intensive livestock production systems, the misuse of antimicrobials, inadequate biosecurity, and poor waste management are linked to the spread of resistant strains that can be transferred to humans through the food chain [11]. Post-mortem can cause biochemical changes, which affect tenderness, water-holding capacity, shelf life, sensory variability, and the risk of adulteration and labeling fraud [19]. Among other factors, quality assurance issues contribute to economic losses and distrust among consumers. There are also pressing environmental sustainability issues, as livestock production is resource-intensive in terms of land use, feed, and water use, as well as greenhouse-gas emissions, nutrient runoff, and pressures on biodiversity [1]. Safety risks related to the production and consumption of animal-source foods have several dimensions highlighted in Figure 2. However, the available literature on reported outcomes of safety interventions has great variability due to the variation in species, production systems, and methodological approaches. Therefore, this review provides a critical assessment of such findings and an explanation of factors that lie beneath such variations. These impacts vary markedly according to species, production system, and region. The interrelationship among microbial hazards, chemical residues, supply-chain integrity, and environmental externalities sets up a highly complex, interlinked problem matrix that requires intervention through integrated technology, management, and policy approaches.

**Figure 3:**
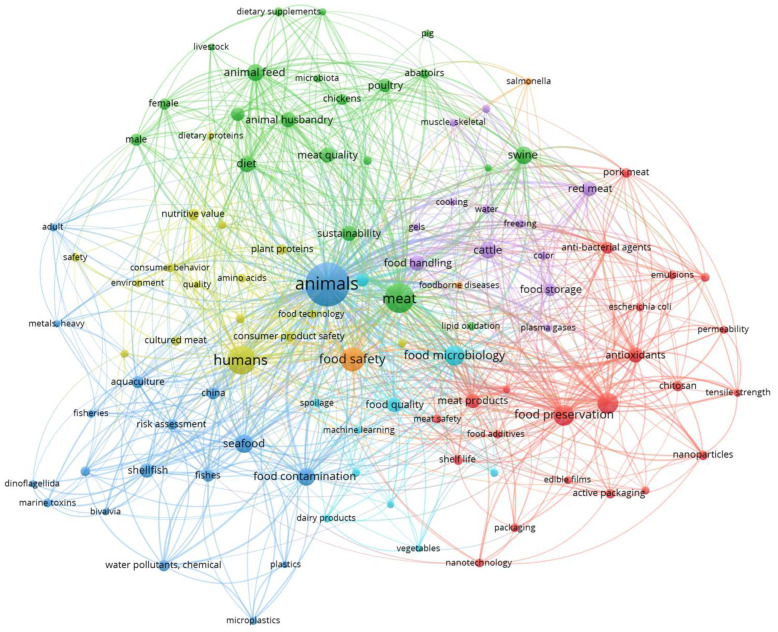
The keyword co-occurrence network, which is generated from the literature published over the past few years on meat quality, safety, and sustainability.

This review synthesizes recent developments in meat safety, enhancing quality, and sustainability, incorporating technological, nutritional, and policy viewpoints into one framework. The relevance of this work is such that, while many single technologies have been reviewed, only a few comprehensive reviews have addressed such innovations in regard to public health consequences, practical viability, and sustainability to translate into practice. Further, by comparing heterogeneous findings among studies, identifying discrepancies, and emphasizing the limitations of each approach that prevent industrial acceptance, the review provides the necessary input to drive further research, regulatory policy, and technology adoption within the meat industry. In addition, a large number of publications have appeared in the past years, as illustrated in Figure 3. These works express the importance of the topic. Though meat safety and enhancement of quality innovations and policies could be reviewed separately, these areas are intrinsically correlated within the current concept of a meat system. Technological interventions aimed at improving microbial safety commonly have effects on either product quality or regulatory acceptability, and vice versa, while sustainable solutions must also comply with safety standards and consumer expectations. For this reason, the present review considers an integrated approach that synthesizes the latest advances in the aforementioned four domains, filling a literature gap where these technologies have so far been discussed singly. This integrated perspective thus allows a more realistic assessment of how new interventions interact across the entire meat value chain and how they would ultimately affect public health.

## 2. Technological Advances in Meat Safety

### 2.1. Traditional vs. Modern Approaches to Meat Safety

Ensuring meat safety has evolved from traditional inspection-based practices to sophisticated, science-driven systems [12]. Historically, meat safety relied on visual inspection of carcasses, organ examination, and sensory evaluation, with microbial testing limited to culture-based methods [20]. In addition, these methods were only sensitive to gross pathological abnormalities and spoilage [20]. They were insensitive to subclinical infections or low levels of contamination, resulting in an opportunity for outbreaks of pathogens such as *Salmonella* or *Listeria monocytogenes* [21]. Traditional preservative techniques, such as smoking, salting, drying, and fermentation, were used to extend shelf life; however, they were unreliable due to inconsistencies in technique and environmental conditions [22].

Modern methods emphasize prevention and risk-based systems that incorporate science, regulation, and technology to enhance safety and security. The HACCP models focus on identifying the hazards in the production chain and adopting control measures at the critical control points [23]. In addition, regulatory authorities of other parts of the world, such as USDA-FSIS and EFSA, have also promoted the paradigm shift by bringing all of them under standardized safety protocols. Furthermore, the introduction of microbial risk assessment models, predictive microbiology, and continuous monitoring systems helps the processors to predict the pollution level instead of being simply responsive to it [24]. Technological innovations also revolutionized the safety management [25]. Mainly, non-thermal processing technologies [26], sophisticated packaging systems [27,28], and automated inspection [12] have been integrated with traditional processes. For instance, high-pressure processing and cold plasma inactivate the pathogens, which has no adverse impact on the quality of meat [29]. These are intensity-dependent, which would lead to some undesirable chemical byproducts or alter the sensory traits with improper intensities. Their scalability is also dependent on regulatory constraints and consumer acceptance. Blockchain and AI-based digital tools offer traceability and predictive monitoring opportunities for a quick response in contamination cases [30]. Since the management of the food system is shifting away from inspection-based practices to more proactive and data-driven approaches, a number of new techniques are established to enhance meat quality and safety in recent times [31]. Microbiology, engineering, and regulatory science now go hand in hand to ensure a reliable global meat supply [32]. These techniques are being utilized to maintain food safety and gain more consumer confidence. However, despite these benefits, their scalability, data governance requirements, and interoperability challenges among the supply chain actors are limiting its real-world adoption.

### 2.2. Microbial Detection and Control

Microbial contamination is the primary risk to meat safety, as pathogens cause widespread foodborne diseases and hospitalizations [33]. Traditional techniques of detection, including culture-based plating, selective media, and biochemical tests, remain a standard in most laboratories, as they are reliable for isolating viable pathogens such as *Salmonella*, *E. coli O157:H7*, and *Listeria monocytogenes* [34]. These methods, however, have a long incubation time, and in some cases, can be affected by not detecting low-level or stressed organisms; therefore, they might not be instrumental in making quick decisions in industrial supply chains [35]. However, rapid diagnostic tools also face limitations such as inhibitor effects from fat and heme, requiring rigorous validation before industrial use. Implementation is further constrained by cost, equipment needs, and inconsistent performance across different meat matrices.

Molecular, immunological, and predictive microbial control is an aspect of modern control [36]. Immunoassays, such as ELISA and lateral flow tests, quickly screen for a particular pathogen or toxin [37]. PCR-based techniques, such as quantitative and multiplex PCR, can detect and quantify in a few hours, offering high specificity and sensitivity [38]. Metagenomics and next-generation sequencing enable the study of microbial communities and the comprehensive identification of antibiotic resistance genes, thereby helping to prevent risks [39].

The control strategies for microbial contamination and meat spoilage need interventions both at the pre- and post-slaughter stages. Biosecurity, vaccination [40], probiotics [41], and feed additives minimize pathogen colonization in live animals. Hot water washes, organic acid sprays, and steam pasteurization are some interventions that minimize carcass contamination during the processing phase [42]. Non-thermal methods such as high-pressure processing and cold plasma methods provide an added advantage of microbial inactivation without any compromise in sensory and nutritional properties [43]. These multi-hurdle strategies that combine these approaches provide a cumulative protection, shifting to proactive control from reactive detection. Most laboratory findings on natural antimicrobials and LAB cultures have yet to be validated under commercial processing conditions, where stability, cost, and sensory impacts may differ significantly.

#### 2.2.1. Quick Molecular Diagnostics (PCR, qPCR, DNA Barcoding)

The method of rapid molecular diagnostics has become a transformative tool for detecting pathogens and authenticating meat [44]. PCR (polymerase chain reaction) enables the amplification of specific DNA strands, allowing for the identification of *Salmonella*, *Listeria*, and pathogenic *E. coli* within just a few hours [45]. The load of pathogens is highly estimated by quantitative PCR (qPCR), which can be used in compliance monitoring and risk assessment. Multiplex PCR has the advantage of detecting multiple pathogens simultaneously, thereby enhancing efficiency in high-throughput applications [46].

DNA barcoding is finding more and more applications in terms of species authenticity and mislabeling prevention, which are essential for allergen management, regulatory compliance, and fraud prevention [47]. Mitochondrial COI and other genetic markers of short standardized lengths are used to accurately identify species, even when dealing with processed meat products [48]. Combination with high-throughput sequencing enables the simultaneous use of multiple samples, providing end-to-end monitoring of supply chains.

Real-time on-site testing can be enabled through portable polymerase chain reaction devices, microfluidics, and automation, reducing the time to intervention [49]. These instruments allow for proactive hazard management, rapid responses in case of contamination, and facilitated traceability by AI-based analysis and biosensors. The use of molecular diagnostics in formal testing schemes controlled by authorities is another indicator of reliability and increased global acceptance. Current accelerated molecular technology has changed the traditional meat safety paradigm from detection at the end-product stage to proactive, data-driven action plans for enhancing the health status and quality of the supply chain. In spite of these advantages, research reports inconsistent sensitivity of detection within different matrices of meat. These are attributed to variations in sample preparation, inhibitors, and primer specificity. The inconsistencies so reported also express the need for a uniform protocol that ensures reliable application in industrial settings.

#### 2.2.2. Biosensors, Nanotechnology, and AI-Based Tools

Biosensors, nanotechnology, and artificial intelligence (AI) have become practical tools for enhancing meat safety through the real-time, sensitive, and rapid detection of microbial and chemical risks [50]. Biosensors are a combination of biological detection devices, e.g., antibodies, enzymes, or nucleic acids, with a transducer that converts these detector molecules into measurable outputs in the presence of a target analyte [51]. They have found use in meat safety to identify pathogens such as *Salmonella*, *Listeria monocytogenes*, and *E. coli O157:H7* within minutes to hours, much faster than the response time of traditional cultures [52]. Recent advancements in electrochemical, optical, and piezoelectric biosensors have resulted from technological changes that have given these biosensors special benefits in terms of sensitivity, specificity, and portability.

Nanotechnology has helped enhance biosensors’ performance by increasing the surface area, amplifying the signal, and increasing the target binding capacity [53]. The detection sensitivity for low-level contamination of microbes is also enhanced due to the use of gold nanoparticles and carbon nanotubes [54]. Meanwhile, the delivery systems can be nanoscale to control the antimicrobial agents within the packaging [55]. The active detection of nanomaterials is also under study, and variations in color, fluorescence, or electrical conductivity are used to detect the presence of pathogens or compounds that lead to spoilage [56].

Artificial intelligence is an adjunctive technology that processes the vast amounts of data collected by imaging systems [57]. Machine learning algorithms can identify trends in the proliferation of microbes, hotspots of contamination, or spoilage in the production, storage, and distribution of food [58]. This multidimensional biosensor, nanotechnology, and AI strategy will enable quicker detection of the problem, reduce human error rates, and allow for real-time monitoring of the meat supply chain. That will have extensive and positive effects on safety performance. However, performance results from the literature are not consistent. Biosensors tend to have high accuracy in controlled laboratory environments, but are less sensitive in real-world settings because of the differing humidity and temperature. These inconsistencies, therefore, mean that additional validation is needed under commercial situations.

#### 2.2.3. Traceability Technologies and Blockchain

Traceability has become a modern concern in meat safety, and flexibility and accountability are as detailed as possible from farm to fork [59]. Blockchain technology supplies a decentralized registry of all transactions that cannot be altered, tracking all transactions, movements, and steps throughout the meat supply chain [60]. Each block contains validated information of origin, slaughter date, processing conditions, and transport information, etc., which cannot be changed retrospectively; hence, fraud, mislabeling, and document errors are lessened.

The traceability systems enable quick recalls; they are able to track the origin of contamination, thus reducing the extent of an outbreak by a great proportion [61]. Temperature, humidity, and storage conditions can be tracked when integrated with sensor networks and IoT devices, and the stakeholders can be informed in case there is a deviation that may pose hazard [62]. Also, blockchain aids in regulatory compliance and consumer confidence since stakeholders can view the source and various parameters of meat quality online using digital channels [63].

**Figure 4:**
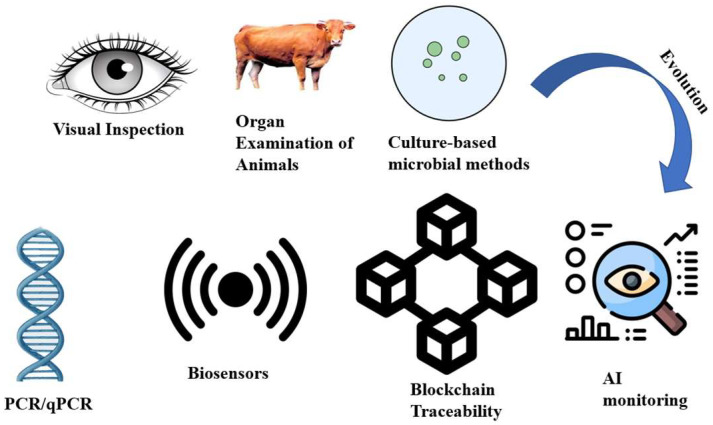
Traditional to advanced evolution of meat safety technologies.

These technologies are increasingly being adopted globally, and pilot projects and commercial rollouts show that they enhance foodborne risk reduction, efficiency of recalls, and consumer confidence. For instance, huge beef and poultry producers have joined forces with technology companies to begin deploying blockchain-based solutions to integrate QR codes, radio-frequency identification tags, and cloud-based databases [64]. The approach ensures that the pathogen and animal welfare standards, feed sources, and organic certification can be traced. Traceability systems and blockchain are novel approaches to meat safety in that they promote end-to-end transparency and thus help expedite decision-making for favorable health outcomes among the population. Figure 4 depicts the movement from traditional meat safety approaches to newer, information-based systems. Blockchain-enabled traceability has emerged as a particularly promising approach, but its outcomes have also varied significantly between studies. These disparities largely relate to differences in infrastructure, stakeholder engagement, and the degree to which existing systems can support the addition of new technologies. Such variation underlines the necessity for standardized data formats and more economy-friendly methods of implementation that would allow the wider diffusion of this technology.

**Figure 5:**
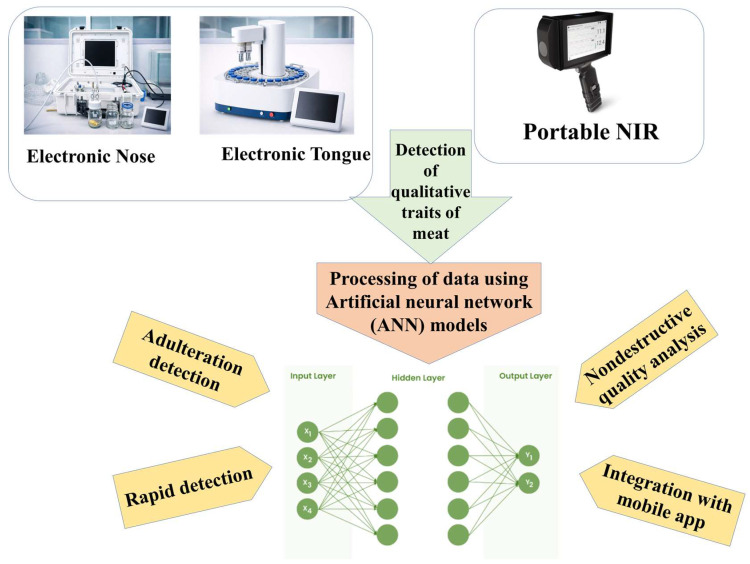
Process of adulteration detection using biomimetic analysis and ANN models.

Several key methodologies are followed for the detection of meat adulteration, comprising the TBARS test and other verification tests. All these traditional methods were more time-consuming and involved higher expenses. However, this is an excellent opportunity to use AI-based models for the processing of data and the detection of overall quality parameters of meat. In addition, Figure 5 presents biomimetic analysis for the detection of meat adulteration and the processing of data through ANN models. The processing through the ANN model generally involves acquisition of data from biomimetic sensors, pre-processing of the spectral or chemical signatures, extraction of features, and classification with the help of a trained multilayer perceptron. The ANN learns patterns for authentic versus adulterated meat by adjusting weights through backpropagation.

### 2.3. Processing and Preservation Technologies

The current development of processing and preservation technologies has significantly enhanced the microbial safety, quality, and shelf life of meat products [65]. Conventional preservation techniques, including salting, smoking, and drying, are being gradually supplemented by new interventions that enhance efficacy while also positively affecting sensory and nutritional characteristics. Non-thermal methods, including high-pressure processing (HPP), cold plasma, and irradiation, enable the inactivation of pathogens and spoilage organisms without altering protein structure, flavor, or color [66]. HPP is one of the best examples among various methods, which has been proven to maintain tenderness and moisture content in ready-to-eat meats, inactivates *Listeria* and *Salmonella*, and is especially applicable to high-value products [67]. The methods used in thermal interventions are not outdated, particularly when combined with proper temperature readings and automation, which serve as means to reduce over-processing and prevent quality loss [68]. There is also the use of hurdle technologies, which is a combination of multiple preservation techniques, such as pH adjustment, low water activity, gentle heating of small amounts, and natural preservatives that offer synergistic protection against microorganisms, thereby extending the shelf life [69]. The new methods focus on bio-preservatives, such as bacteriocins, protective cultures, and plant-based antimicrobials [70]. The interventions to control pathogens are also used to meet the clean-label requirements of consumers [71]. Antimicrobial films and oxygen scavengers are active technologies used to preserve meat, which, in addition to moderating the condition of food preservation, promotes further resistance to microbial growth [72]. The integration of these technologies in processing and preservation enables the meat industry to produce safer meat with a longer shelf life, reduced spoilage, and increased consumer acceptance, indicating a shift toward more evidence-based, scientifically optimized management of meat safety.

#### 2.3.1. Cold Plasma, Ozone, and Irradiation

Cold plasma, ozone treatment, and irradiation are also non-thermal decontamination techniques that have been readily adopted due to their ability to inactivate pathogens in meat without affecting its sensory and nutritional characteristics [73]. Cold plasma is a reactive gas composed of ionized components, including electrons, ions, and radicals, which can destroy the membranes and DNA of microbial cells [74]. *Listeria monocytogenes*, *Salmonella* spp., and *Escherichia coli* on meat surfaces respond to cold plasma, with often dramatic improvements observed in just a few minutes after exposure [75]. Moreover, cold plasma can be applied at near-ambient temperatures, thereby minimizing the impact of cold plasma on meat texture, color, and flavor compared to conventional thermal processing [76].

Ozone is a highly effective oxidizing agent that is applied in either gaseous or aqueous form to reduce microbial loads on processed meat and carcasses [77]. Its antimicrobial activity is attributed to the oxidation of cell walls and intracellular components, resulting in the elimination of microbes [78]. The use of ozone systems has been effective in controlling bacterial and viral levels during chilling, washing, and packaging processes. The technology is environmentally friendly and leaves no residual effects; however, it must be closely monitored to minimize the potential oxidative impact of lipid exposure and preserve shelf life.

A practical and proven method of decontaminating meat is through the use of irradiation (with gamma rays, electron beams, and X-rays) [79]. It helps kill bacteria, viruses, and parasites, and also increases shelf life by reducing the amount of spoilage microorganisms. Their use is approved by regulatory bodies such as the U.S. FDA and Codex Alimentarius, provided the recommended doses are used [80]. Traditionally, consumer acceptance has been a challenge; however, awareness and confidence in irradiated products have increased through education and labeling. These are non-thermal interventions and other preservation methods that offer a proactive, evidence-based approach to improving meat safety without compromising product quality.

#### 2.3.2. Directly Related to the Product Are Natural Preservatives and Bioprotective Cultures

Natural preservatives and bioprotective cultures have become the subject of research due to consumer demand for minimally processed meat products that are clean-label [81]. Plant-based compounds, including essential oils, phenolic extracts, and organic acids, possess antimicrobial properties and antioxidants that prevent food spoilage and inhibit the growth of pathogenic microorganisms [82]. Rosemary and oregano extracts have been shown to help prevent the development of *Listeria* and *Salmonella* in ready-to-eat meats, as well as lipid oxidation [83]. There are two significant benefits of these blends. First, they contain no chemical additives; secondly, their shelf life is increased without compromising nutritional value.

The decisive characteristics of bioprotective cultures include competitive exclusion, acidification, and bacteriocins (small antimicrobial peptides also active against pathogens, such as *Listeria monocytogenes*), which are typically produced by lactic acid bacteria (LAB) [84]. LAB added to fermented sausages and ready-to-eat meats enhances microbial safety, taste, and texture; thus, safety interventions can be used together with sensory quality. Using natural antimicrobials in combination with bioprotective cultures is a multi-barrier approach that offers complementary mechanisms of microbial control, aligning with consumer expectations for natural and unprocessed products.

These processes are becoming part of a global approach to microbial safety, where they are used together with conventional and non-thermal decontamination processes in industrial meat processing. To maximize the efficacy of these compounds while minimizing the sensory and nutritional effects, constant research is being conducted to optimize the dosage, mode of delivery, and synergies with packaging technologies.

#### 2.3.3. Safety Innovation Through Active Packaging

One of the most important aspects of meat storage is active packaging that facilitates extending the shelf life or increasing the safety of meat during storage [85]. Active packaging is a new technology that reacts with the product or with the environment to inhibit microbial growth [86]. Active packaging systems utilize oxygen scavengers, moisture absorbers, and antimicrobial agents (such as silver nanoparticles or natural extracts) to create a controlled microenvironment that inhibits the growth of microorganisms in the meat, thereby preventing spoilage.

Smart packaging is another technology that enables the monitoring of product conditions and the transmission of information to processors and consumers [87]. Smart packaging technology utilizes RFID, sensors, and indicators to consistently record real-time temperature, microbial activity, and pH levels [88]. Such systems can alert supply chain managers or consumers when occurrences of deviations that could affect product safety occur. Furthermore, prompt action could be taken to recall the products.

A combination of active and intelligent packaging can enhance the process of food safety by minimizing microbial hazards and increasing transparency.

#### 2.3.4. Antibacterial Films and Biodegradable Materials

The use of antimicrobial films and biodegradable substances can also support meat safety in two aspects: first, they reduce the risk of microbial contamination, and second, they can address environmental sustainability concerns [89]. In traditional plastic-based packaging, plastic has a negative environmental impact since it is not readily biodegradable and has a large carbon footprint [90]. Biodegradable polymers, such as polylactic acid (PLA), polylactic acid derivatives, cellulose, and starch-based materials, are also considered sustainable materials that can be safely degraded in a natural environment without negatively impacting the packaging’s functionality [91].

The inclusion of antimicrobials in these films also increases the safety of meat [92]. The surface of meat itself can be protected against microbial growth with natural antimicrobials, such as essential oils, chitosan, and bacteriocins. *Listeria monocytogenes* and *Escherichia coli* can be controlled by chitosan-based films in fresh and processed meat products [93]. The additional advantage of antimicrobial films is that the insecticide remains active during storage and distribution, thereby maintaining the product’s high quality throughout its extended shelf life.

These innovations align with consumer demands for products featuring long-lasting and safe meat packaging that meets regulatory standards. Extensive research is being conducted to optimize mechanical strength, barrier properties, and controlled release rates, to achieve functional performance comparable to that of traditional plastics. The antimicrobial-biodegradable packaging in modern meat processing demonstrates a comprehensive safety and sustainability strategy, microbial assurance, and an eco-friendly nature.

### 2.4. Physicochemical Quality Improvement

Physicochemical quality is the primary determinant of the consumer acceptance of meat and its nutritional value [94]. Meat qualities, such as tenderness, juiciness, and overall acceptability, are influenced by pH, water-holding capacity, color, and protein solubility. Alteration of these properties through changes in processing conditions is achieved by processing the food by marinating or the addition of additives. Phosphates and added salt can increase the water-holding capacity, reduce cooking losses, and maintain the texture [95]. Additionally, non-thermal processes such as high-pressure processing (HPP) [96] alter physicochemical characteristics by controlling protein structure, enhancing digestibility, and inactivating microorganisms that cause food spoilage. It is possible to monitor these parameters spectrophotometrically, rheologically, and by imaging, allowing processors to maintain the same quality standards throughout the batch and satisfy industry or customer demand [97].

#### 2.4.1. Modifications (e.g., Transglutaminase) with Enzymes

Interventions based on enzymes, particularly microbial transglutaminase (MTGase), have enhanced meat texture and functionality [98]. The cross-linking of glutamine and lysine residues in proteins is facilitated by the MTGase catalyst, which enhances the gelation, water-holding capacity, and cohesiveness of restructured meat products [98]. This enables the fabrication of patties, sausages, and meat analogs with consistent texture and reduced mechanical processing. Enzymatic modification also allows the incorporation of plant-based proteins or fillers without compromising the desirable mouthfeel and chewiness [99]. Research has shown that the correct levels of MTGase can be used to enhance tenderness and juiciness without compromising color or taste [100]. The use of transglutaminase also raises regulatory and allergenicity considerations that influence consumer acceptance. Proteolytic enzymes, such as papain or bromelain, make meat tender by partially breaking down connective tissue, particularly in older or more difficult-to-eat cuts [101]. These enzyme-driven solutions provide targeted enhancement of meat quality with high accuracy, relevant to consumer demands, minimize processing in their products, and are additive-aware.

#### 2.4.2. Reduction in Salt and Fat Strategies

High concentrations of sodium and saturated fat in meat products have become a significant health concern in society, and efforts to reduce them without compromising sensory attributes have been explored [102]. Replacement of salt with a partial amount of potassium chloride, calcium salts, or naturally occurring flavor enhancers is widely used to reduce sodium content. Reduction measures include using less fat in meat products, employing structured emulsions, or replacing products with plant-based oils or fibers to maintain juiciness and mouthfeel [103]. Microencapsulation of fat substitutes helps maintain texture and moisture during the cooking process [104]. In addition, transglutaminase and/or enzyme-assisted restructuring are used to help reduce fat while maintaining the structure and mouthfeel as a unit [98]. Combining these strategies enhances the quality of meat products, meeting consumer taste, tenderness, and acceptance criteria.

#### 2.4.3. Ingredients (Plant Proteins, Fibers, Bioactive Compounds): Functional

Enrichment with functional ingredients in meat products is a recent development, particularly in enhancing the nutritional and textural value of the food [105]. To incorporate the functional ingredient, several methods are available, with restructured meat formation being one of the most effective ways to combine functional ingredients [106]. Additionally, the inclusion of antioxidant agents in the hybrid formulation can protect the hybrid product against lipid oxidation and color degradation. Moreover, bioactive compounds such as polyphenols or carotenoids can also be added to hybrid meat products as functional ingredients [107]. These functional ingredients can also enhance emulsification, gel formation, and texture in low-fat restructured meat products [98]. Galani et al. [108] demonstrated that incorporating soy protein isolate and pea protein into meat formulations significantly enhances emulsion stability and reduces cooking loss compared with control samples. Furthermore, Mishra et al. [109] stated that the addition of inulin or citrus fiber has the ability to increase the water-holding capacity and improve texture firmness without negatively affecting sensory acceptance. Furthermore, Schilling et al. [110] stated that rosemary and green tea extracts have the ability to protect the meat against oxidation and preserve meat quality.

The addition of plant protein to animal-based protein, as seen in the formulation of hybrid meat products, has several benefits. Researchers have suggested a synergy between plant-based proteins and enzymatic or hydrocolloid interventions, which enables the development of meat products that are balanced in terms of health, functionality, and sensory attributes.

## 3. Advances in Technology for Enhancing Meat Quality and Sensory Attributes

### 3.1. Improvements in Sensory Quality

Sensory Quality (encompassing the sensual aspects of taste, smell, and touch) is also a critical consumer acceptance criterion [111]. These require technological interventions that would improve them without spoiling the nutritional integrity [12]. They are promoted by enzymes, salts, or flavor enhancers, which create tenderness and juiciness [98]. Antioxidants and natural extracts help maintain color and prevent the off-flavors that form due to lipid oxidation [112]. Parameters in the processing are also optimized and regulated, such as temperature, humidity, and time, to minimize protein denaturation and further reduce moisture loss. Moreover, restructured meat products utilize binding agents and functional proteins to achieve a homogeneous texture and mouthfeel [98]. Historically, these qualities have been evaluated by human panels of experts; technological progress in sensory evaluation devices, such as electronic nose and electronic tongue, also enables the objective evaluation of these qualities. These measures make meat products both healthy and delicious.

### 3.2. Optimization of Flavor, Texture, and Tenderness

The key quality traits shaped by muscle composition, postmortem aging, and processing are flavor, texture, and tenderness [113]. Controlled aging at controlled temperature and humidity enhances tenderness by increasing the enzymatic breakdown of myofibrils in the muscle, and flavor precursors are generated through Maillard reactions and lipid oxidation [114]. New processes, which include tenderization using enzymes, reduced salt marinade, and cross-linking of proteins by transglutaminase, enhance chewiness, cohesiveness, and juiciness [98]. Hydrocolloids and plant-based proteins are functional ingredients that can improve texture without compromising taste [115]. These biochemical and technological strategies are combined with optimization strategies to offer meat products that cater to the food and nutritional needs. Despite their benefits, reported outcomes vary considerably across studies. For example, enzymatic tenderization may improve texture in some muscle types but cause over-softening in others. At the same time, the incorporation of plant protein can enhance juiciness yet negatively affect flavor, depending on the formulation. Such variability underscores the need for more systematic comparative studies.

### 3.3. Artificial Intelligence Sensory Analysis Tools

Sensory quality measurement of meat products using artificial intelligence (AI) is becoming increasingly common [12]. The data generated by electronic noses, electronic tongues, and image-imaging systems are transformed into data that is subject to machine learning algorithms, which objectively determine flavor, aroma, texture, and color [12]. AI-based models enable the anticipation of consumer perception and the detection of minor defects that can go unnoticed by human panels. The AI-based analysis can reduce variability, accelerate product development, and provide real-time feedback to inform adjustments [116]. Integrating supply chain big data enables the prediction of quality changes throughout storage and distribution. AI complements traditional food sensory analysis to provide standardized, repeatable, and effective quality control for a diverse range of meat products.

### 3.4. Automated Quality Grading Inspection

There is an objective measure of AI-based models in detecting meat quality characteristics (color, marbling, surface defects, and fat distribution) [117]. The combination of high-resolution images, pattern recognition, and AI algorithms can assist the processing plants in sorting and grading the quality of the product being produced very fast [12]. This automation, along with the use of automated production lines, enhances the safety, productivity, and consistency of large-scale meat operations. However, AI-based grading models are sensitive to dataset size, representativeness, and hardware variability, which affects model robustness in real industrial environments.

## 4. Sustainable Meat Production Technology

The world’s meat consumption has strained environmental resources to the limit, underscoring the need for the development of sustainable meat production systems. The transformation of traditional meat systems into eco-friendly, resource-sensitive, and health-conscious products, such as meat analogs [12] or utilizing a production system with a lower environmental impact, e.g., Precision Livestock Farming (PLF) [115]. PLF is one such technology, leveraging the functions of sensors, automated tracking, and data analytics to improve the health, productivity, and welfare of animals, while reducing feeding and water wastage [118]. PLF enables the efficient utilization of resources and a reduced environmental impact by continuously monitoring physiological and behavioral parameters.

Sustainability is also related to resource-efficient feed and water management [119]. These are because the optimal water usage, alternative feeds, and high-nutrition feed compositions not only lower the emission of greenhouse gases, but also swell the feed ratios. In addition to traditional systems, alternative protein technologies are transforming the prospects of sustainable meat [120]. Cultured meat and cellular agriculture offer meat products derived from animal cells, eliminating the need for livestock and thereby avoiding the consumption of vast amounts of land and water [99]. The emission of methane gases generated in conventional farming is also avoided [99]. Furthermore, meat alternatives of plant origin and hybrid products that incorporate a combination of plant-based proteins with a minor portion of animal proteins also offer nutritionally sufficient alternatives, which should have a less drastic ecological footprint [12]. A type of microbial protein is a single-cell protein derived from algae, fungi, or bacteria, serving as a high-efficiency and low-footprint protein source that supplements or partially replaces traditional meat [1]. The sustainability of the circular economy approaches is also associated with the fact that meat by-products are valued, and wastage is minimized [98]. The most promising way to utilize animal resources is to produce protein-rich powders, bioactive molecules, or energy tablets from bones, offal, and blood [121]. Moreover, there is a reduction in the carbon footprint due to the waste management practices and energy processing technologies utilized. The environmental effects can be quantified using quantitative assessment tools, such as Life Cycle Assessment (LCA), allowing for informed decisions on reducing greenhouse gas (GHG) emissions, energy consumption, and water usage across the meat value chain [122]. Reported LCA outcomes vary widely depending on system boundaries and production scale, and several technologies remain conceptual rather than commercially scalable. These technological advances contribute to the establishment of a sustainable and healthy meat industry that meets environmental and population health standards. Furthermore, the technological advances are discussed in Table 1 along with the benefits. However, sustainability outcomes differ widely among published studies due to variations in production scale, energy sources, and LCA methodological assumptions. These inconsistencies underscore the importance of standardized environmental assessment frameworks for ensuring fair comparisons across technologies. The success of emerging technology in the processing and production of meat has enormous implications for human health, food security, safety, nutrition, and consumer acceptance [12]. These innovations have enhanced the credibility of meat as a valuable source of protein by improving its quality and ensuring food safety through the development of new diagnostic technologies, rapid microbial detection methods, and AI-based monitoring systems. Consequently, a reduction in the number of contaminated meats would minimize the risks of foodborne illnesses. Moreover, improved methods of processing, preservation, and packaging are being adopted to slow down the growth and spoilage of microorganisms, thereby providing a safe supply of meat products to consumers.

The reduction in antibiotic usage in livestock can be achieved through close monitoring of pathogens and the application of other preservation techniques [123]. This will reduce the diffusion of resistant bacteria in the food chain, thereby protecting human health and aligning with global public health objectives. Other functional and nutritional benefits can also be obtained, in addition to safety, through technological interventions [12].

Enrichment, e.g., fortification of the meat products with vitamins, minerals, or bioactive compounds to enhance the health profile of the product [98]. Clear labeling, tracking, and information about the benefits of cultured meat, hybrid products, and enhanced preservation methods can be used to foster consumer acceptance of new meat products. Products with definite safety, nutritional, and environmental benefits have higher chances of being adopted by consumers.

**Table 1:** Technological methods and their sustainability benefits in advancing meat production systems.

Category	Technologies/Approaches	Sustainability Benefits	References
Precision Livestock Farming (PLF)	Sensors, AI monitoring, automated health checks	Optimized resource use, improved welfare, reduced emissions	[124]
Feed and Water Efficiency	High-nutrition feeds, alternative feed ingredients	Lower feed waste, improved conversion efficiency	[125]
Cultured Meat and Cellular Agriculture	Lab-grown meat from animal cells	Reduced land and water use, zero livestock emissions	[1]
Plant-based and Hybrid Analogs	Plant proteins, hybrid meat formulations	Lower carbon footprint, reduced environmental impact	[115]
Microbial Proteins	Algae, fungi, bacteria-based proteins	High-efficiency protein production, minimal land/water use	[1]
Circular Economy Approaches	Valorization of by-products, energy recovery	Reduced waste, resource maximization	[121]
Life Cycle Assessment (LCA)	Carbon footprint and environmental impact analysis	Quantifies environmental benefits, guides sustainable decisions	[126]

## 5. Policy, Regulatory, and Ethical Perspectives

Meat production and innovation governance must have robust policies, rules, and ethical systems that ensure meat production is safe, of high quality, sustainable, and trusted by the people. International standards provide uniform guidelines to regulate microbial contamination, chemical residues, and processing procedures, ensuring that meat products are safe to consume. Global organizations, such as the Codex Alimentarius Commission [127], the European Food Safety Authority (EFSA) [128], and the U.S. Food and Drug Administration (FDA) [129], establish safety thresholds, handling procedures, and observatory measures to safeguard public health. Furthermore, these aspects are briefly discussed in Table 2. The processes of certification, labeling, and transparency are becoming more critical in developing consumer trust. HACCP, ISO 22000 [130], and organic or halal labels are some of the certifications that help consumers understand the safety standards and ethical principles in meat production [131]. Labeling, which includes information on sourcing, production methods, and nutrition, enables consumers to make informed decisions and promotes accountability within the industry. Moreover, blockchain or IoT-based traceability systems make the process more transparent by tracking meat from the farm to the fork.

Ethical concerns are particularly pertinent to new technologies, such as alternative meats, including cultured meat, hybrid products, and microbial proteins. Current regulatory frameworks do not fully address emerging technologies such as nanomaterials, cultured meat, or AI-based quality assessment, and perceptions of naturalness and socio-economic disparities continue to shape public acceptance. The moral issues revolve around animal ethics, environmental issues, and fair access to technology. Lab-grown or hybrid meat production will decrease the need to rear livestock, aligning with the ethical goal of reducing animals’ suffering and their environmental footprint. Policymakers must strike a balance between innovation and societal values, considering issues of naturalness, cultural acceptance, and fairness in the distribution of new food technologies.

Regulatory adherence, open practices, and ethical governance are crucial to ensuring that technological innovations in meat production enhance safety, quality, and sustainability, thereby maintaining trust and acceptance in society. Despite the rapid advancement of emerging technologies, several obstacles limit their real-world implementation. Many innovations, such as cultured meat, microbial proteins, and advanced packaging systems, remain constrained by high production costs and limited scalability, making commercial adoption challenging for industry stakeholders. Furthermore, regulatory frameworks for novel foods differ widely across countries, and the absence of clear approval pathways can delay market entry or restrict international trade. These economic and policy barriers highlight the need for harmonized regulations, cost-reduction strategies, and large-scale validation studies before these technologies can achieve meaningful market penetration.

## 6. Future Prospects and Research Directions

The transformation in the meat industry will be revolutionary due to new technologies and digitalization that change current patterns of production, processing, and consumption. Putting together AI, IoT, and Big Data in meat supply chains opens new opportunities for real-time tracking of products, predictive analytics, and decision-making based on this input [30]. AI-based models can make predictions about spoilage, optimize the parameters of the treatment process, and further enhance quality control, while IoT devices can facilitate traceability, environmental monitoring, and resource management through automation. Big Data analytics is able to combine data on production and consumption with health data, which enables decisions on sustainable processes and policies [117].

There is also an emerging trend in the use of new technologies in personalized nutrition and meat products [6]. Using genomics, metabolomics, and AI, meat products could be tailored to personal nutritional needs, preferred diets, or specific medical needs [98]. This will result in greater satisfaction for the consumer while reducing some of the major public health problems, including malnutrition, obesity, and micronutrient deficiencies. Alternatives: Hybrid, fortified, and cultured meat are also alternatives that provide more choice for a person in their eating [99].

The key challenge here is balancing technological development with accessibility and affordability. While it is true that increased processing and alternative protein technologies have great environmental and health benefits, cost, scalability, and consumer acceptance are major barriers to overcome [12]. The work going forward must be in developing more affordable, scalable, and accessible technologies that can be deployed by industrial producers and in low-resource settings. Policies and approaches for engaging with people are also required so that innovations come into practice on fair terms, not to sacrifice safety, quality, and ethics. Cross-cutting bottlenecks—which include needs for validation, regulatory alignment, scalability limitations, and socio-economic constraints—need to be tackled simultaneously so that these technologies see a real translation into reality.

## 7. Conclusions

Technological innovations in the production and processing of meat are revolutionizing the industry, improving not only the safety, quality, and sustainability of meat but also tackling significant concerns for health among the population. Among such innovations, only a few have demonstrated feasibility in the near term, while others will require extensive validation and regulatory harmonization before scaling can occur. The review outlines, critiques, and synthesizes the current trends, showing how modern technologies can enhance meat safety by facilitating faster microbial detection, utilizing biosensors, and implementing control systems with the use of artificial intelligence, which help reduce the risk of foodborne diseases and mitigate the issue of antimicrobial resistance. Advanced preservation and processing techniques that have extended the shelf life of meat foods and maintained their sensory qualities include high-pressure processing, cold plasma, smart packaging, and the use of natural preservatives that improve the overall quality of meat.

Sustainability and future protein alternatives also focus on sustainable practices, resource-efficient feed and water consumption, and circular economy approaches that minimize environmental impact and optimize resource utilization. The review discusses promising alternatives, including cultured meat, plant-based products, hybrid products, and microbial proteins. These alternative proteins have the potential to reduce greenhouse gas emissions, land use, and water consumption, while meeting the growing demand for protein. With regard to assessing the influence of such innovations on the environment, LCA is indeed a robust tool for making more informed decisions on sustainability.

The effect on public health is significant, from safety to nutritional value, functionality, and consumer welfare. Other key variables that give the consumer trustworthiness and social acceptability include transparent certification, labeling, traceability systems, and ethical considerations on the production of alternative meat. More research needs to be performed to integrate AI, IoT, and Big Data analytics for seamless supply chains, real-time quality track, and customized nutrition options. It should be affordable and available to all populations and regions, with equitable benefits. Moreover, the literature reveals diverse and sometimes conflicting results regarding microbial reduction, sensory improvements, and environmental impact across technologies. These inconsistencies highlight the need for harmonized validation methods, long-term studies, and cross-disciplinary collaboration to ensure the reliable implementation of these measures.

## Figures and Tables

**Table 2:** Governance frameworks, regulatory authorities, and ethical considerations shaping meat safety, quality, transparency, and sustainability.

Aspect	Key Measures/Technologies	Impact on Meat Safety, Quality, and Ethics	References
Global Regulations	Codex Alimentarius, EFSA, FDA, HACCP	Standardizes safety protocols, reduces contamination risk	[127,128,129]
Certification and Labeling	ISO 22000, organic, halal, traceability systems	Increases transparency, informs consumer choice, and builds trust	[131]
Ethical Considerations	Cultured meat, hybrid products, and animal welfare policies	Minimizes animal suffering, reduces environmental impact, and promotes equitable access	[98]
Transparency and Traceability	Blockchain, IoT-based supply chain monitoring	Ensures accountability from farm to fork, prevents fraud	[30]

## Data Availability

No new data were created or analyzed in this study.
